# *NOTCH1* PEST domain variants are responsive to standard of care treatments despite distinct transformative properties in a breast cancer model

**DOI:** 10.18632/oncotarget.28200

**Published:** 2022-02-16

**Authors:** Karen Cravero, Morgan V. Pantone, Dong Ho Shin, Riley Bergman, Rory Cochran, David Chu, Daniel J. Zabransky, Swathi Karthikeyan, Ian G. Waters, Natasha Hunter, D. Marc Rosen, Kelly Kyker-Snowman, W. Brian Dalton, Berry Button, Dan Shinn, Hong Yuen Wong, Joshua Donaldson, Paula J. Hurley, Sarah Croessmann, Ben Ho Park

**Affiliations:** ^1^The Sidney Kimmel Comprehensive Cancer Center, The Johns Hopkins University School of Medicine, Baltimore, MD, USA; ^2^Division of Hematology, Oncology, Department of Medicine, Vanderbilt University Medical Center and The Vanderbilt-Ingram Cancer Center, Nashville, TN, USA; ^*^These authors contributed equally to this work

**Keywords:** NOTCH1, TNBC, breast cancer, PEST

## Abstract

Activating variants in the PEST region of *NOTCH1* have been associated with aggressive phenotypes in human cancers, including triple-negative breast cancer (TNBC). Previous studies suggested that PEST domain variants in TNBC patients resulted in increased cell proliferation, invasiveness, and decreased overall survival. In this study, we assess the phenotypic transformation of activating *NOTCH1* variants and their response to standard of care therapies. AAV-mediated gene targeting was used to isogenically incorporate 3 *NOTCH1* variants, including a novel TNBC frameshift variant, in two non-tumorigenic breast epithelial cell lines, MCF10A and hTERT-IMEC. Two different variants at the *NOTCH1* A2241 site (A2441fs and A2441T) both demonstrated increased transformative properties when compared to a non-transformative PEST domain variant (S2523L). These phenotypic changes include proliferation, migration, anchorage-independent growth, and MAPK pathway activation. In contrast to previous studies, activating *NOTCH1* variants did not display sensitivity to a gamma secretase inhibitor (GSI) or resistance to chemotherapies. This study demonstrates distinct transformative phenotypes are specific to a given variant within *NOTCH1* and these phenotypes do not correlate with sensitivities or resistance to chemotherapies or GSIs. Although previous studies have suggested *NOTCH1* variants may be prognostic for TNBC, our study does not demonstrate prognostic ability of these variants and suggests further characterization would be required for clinical applications.

## INTRODUCTION

Triple negative breast cancer (TNBC) is a subtype of breast cancer that accounts for 15–20% of all diagnosed patients. Traditionally, breast cancer is treated based on the expression of estrogen receptor (ER), progesterone receptor (PR), and/or human epidermal growth factor 2 (HER2) receptor using endocrine and HER2 targeting therapies, respectively. However, patients with TNBC lack expression of these receptors and are therefore limited to standard surgery, chemotherapy, and radiation for treatment options. More recently, immunotherapy has been approved for select patients with TNBC in both the metastatic and early-stage settings. Treatment efficacy is complicated by the heterogenous nature of TNBC. In 2011, Lehmann *et al.* demonstrated TNBC can be classified into distinct subtypes based on gene expression profiles and these molecular differences may dictate response to therapy [1]. These distinct molecular differences combined with the aggressive nature of TNBC have resulted in poor treatment options, increased rates of recurrence and metastases, and decreased overall survival [2–4]. Therefore, there is a pressing need to find alternative treatment options for patients with TNBC. In recent years, clinical studies for TNBC have begun focusing on new targeted therapies, such as poly (ADP-ribose) polymerase (PARP) inhibitors, anti-Trop2 antibody drug conjugates and immunotherapies, along with potential predictive markers for these therapies [5].

One potential target for novel therapies against TNBC is *NOTCH1*. *NOTCH1* variants are found in many cancer types (cBioPortal) suggesting its pathogenic role in cancer growth, invasion, and metastasis [6–9]. Dysregulation of the Notch1 pathway has been frequently identified in different aggressive human cancers and has been shown to play an important role in cancer development [10–14]. Normal Notch signaling is carried out by four different single-pass transmembrane receptors and is essential for cell differentiation. The canonical activation of Notch1 signaling releases the Notch Intracellular Domain (NICD), which translocates to the nucleus upon activation. The γ-secretase (GS) complex is required for cleavage and activation of all four Notch receptors [15]. Aberrant activation of the Notch1 pathway is often the result of PEST domain variants that lead to stabilization of NICD and constitutive activation of Notch1 signaling [16, 17]. Several studies have demonstrated specifically frameshift and truncating PEST domain variants increase the stability and half-life of NICD [18–21].

Notch1 signaling is activated at a significantly higher rate in TNBC compared to other subtypes of breast cancer [18, 22, 23]. Among TNBC patients, Notch1 receptor variants range from missense to frameshift variants and cluster within the PEST domain region (cBioPortal) [24]. Furthermore, TNBC patients with increased Notch1 expression have demonstrated increased aggressive phenotypes and lower median overall survival [25]. In more recent years, the high correlation of aberrant Notch1 signaling and TNBC has gained interest as a potential target for new therapies. In 2015, Wang *et al.* demonstrated γ-secretase inhibitors (GSI) could disrupt Notch1 activation in patient-derived xenografts with PEST domain variants [18]. In the following years, GSIs have been explored in phase I and II clinical trials for breast cancer, pancreatic cancer, and metastatic melanoma [26–28]. While there is still no proven clinical benefit in breast cancer patients, this avenue of therapy may provide a targeted-treatment opportunity for TNBC patients. In our current study we generated and characterized a panel of isogenically modified *NOTCH1* cell lines to characterize the transformative potential of these variants in non-tumorigenic breast epithelial cells and tested the predictive value of these variants to GSIs and current standard of care chemotherapies for TNBC.

## RESULTS

### *NOTCH1* PEST domain variants in non-tumorigenic human breast epithelial cell lines result in NICD Notch1 activation

**Figure 1:**
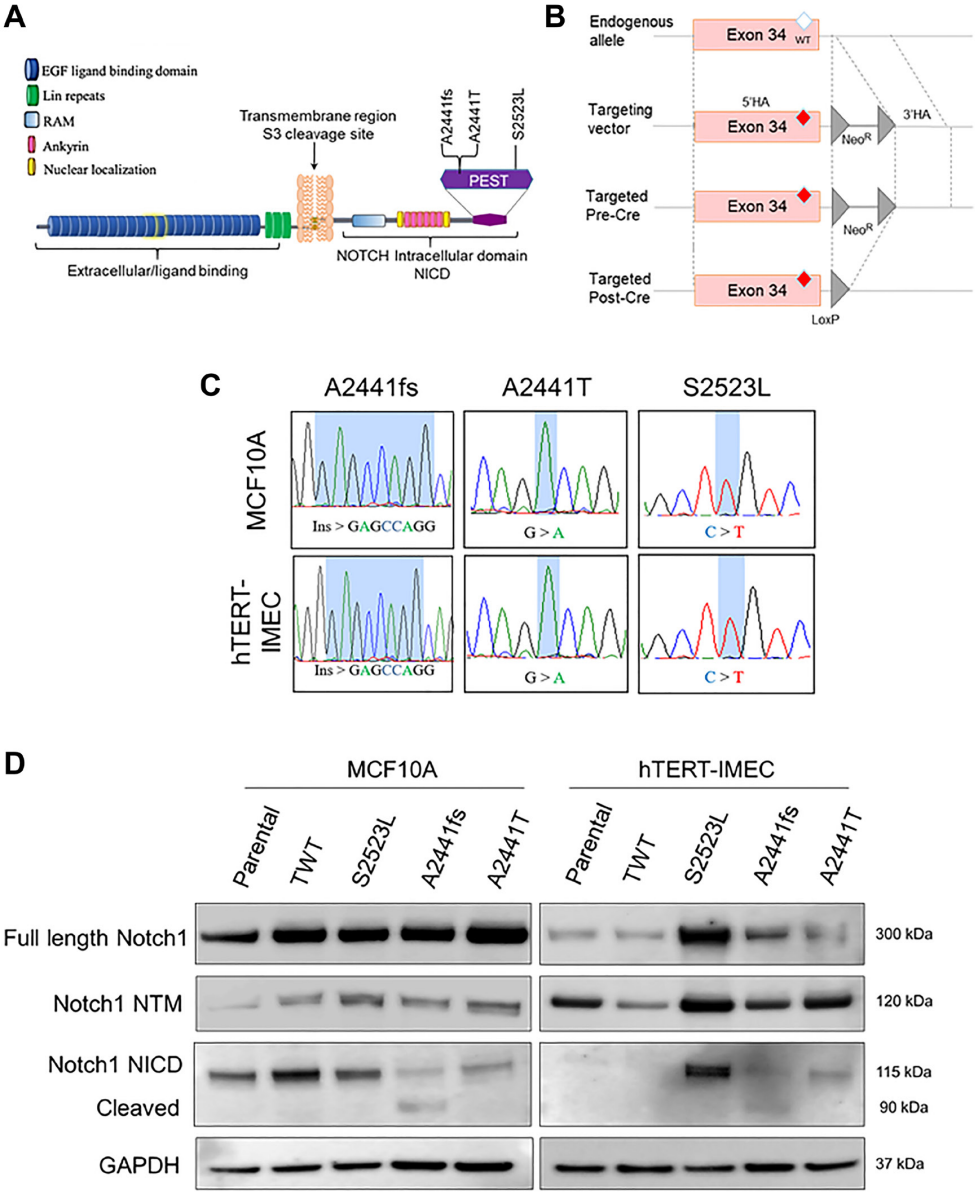
*NOTCH1* isogenic cell panel in nontumorigenic breast epithelial cells. (**A**) Representative *NOTCH1* variants included in the isogenic panel. A2441fs was identified in a tumor board and A2441T and S2523L were identified via cBioPortal. (**B**) Schematic of rAAV-mediated gene targeting of variants in exon 34 of *NOTCH1*. rAAV transduction leads to locus-specific targeting via homologous recombination of the 5′ and 3′ homology arms (HA). After neomycin selection, the isolated clone is subjected to Cre recombinase to excise the neomycin cassette (Neo^R^), resulting in a LoxP site. (**C**) Sanger sequence confirmation of genomic alterations in the *NOTCH1*-PEST cohort for both the MCF10A and hTERT-IMEC panel. (**D**) Immunoblot analysis for Notch1, Notch1 transmembrane (NTM), and Notch1 intracellular domain (NICD) for both the MCF10A and hTERT-IMEC panels. Cleaved version of Notch1 NICD for A2441fs is visualized by the NICD antibody.

Analysis of three publicly available tumor-associated variant databases (TSGene [29], COSMIC [30], and cBioPortal [24]) identified the *NOTCH1* A2441 site as a commonly mutated codon in breast as well as many other cancers (salivary, adrenal, T-ALL, etc). To study the transformative properties of *NOTCH1* variants in nontumorigenic breast epithelial cells, a small cohort of variants were selected for gene targeting. Among the numerous A2441 variants, a frameshift insertion variant (A2441Efs*39, abbreviated A2441fs) and missense variant (A2441T) were selected. In addition to these variants, a PEST domain variant (S2523L) located downstream of the A2441 site was selected to compare transformative properties (Figure 1A). AAV-mediated gene targeting was used to isogenically incorporate the *NOTCH1* variants into two non-tumorigenic breast epithelial cell lines (MCF10A and hTERT-IMEC). In addition to the *NOTCH1* variants, a targeted wildtype (TWT), which underwent the same gene targeting mechanism with a wildtype vector, was generated for both parental cell lines to act as a control. Schematic representation of AAV-mediated gene targeting is shown in (Figure 1B). PCR and Sanger sequencing were done to confirm a single allelic copy of the desired variant in each clone (Figure 1C). As mentioned previously, frameshift and truncating variants in *NOTCH1* result in an extended half-life for the cleaved NICD protein and constitutively active Notch1 pathway. To confirm the novel A2441fs variant had increased cleaved NICD, immunoblot assays for Notch1, Notch1 transmembrane (NTM), and Notch1 intracellular domain (NICD) were performed for both the MCF10A and hTERT-IMEC panels. In both panels, the novel A2441fs variant had a second band present for the NICD immunoblot, representing the higher levels of cleaved Notch1 (Figure 1D).

### *NOTCH1* A2441 variants confer growth factor independence and increased MAPK activity

**Figure 2:**
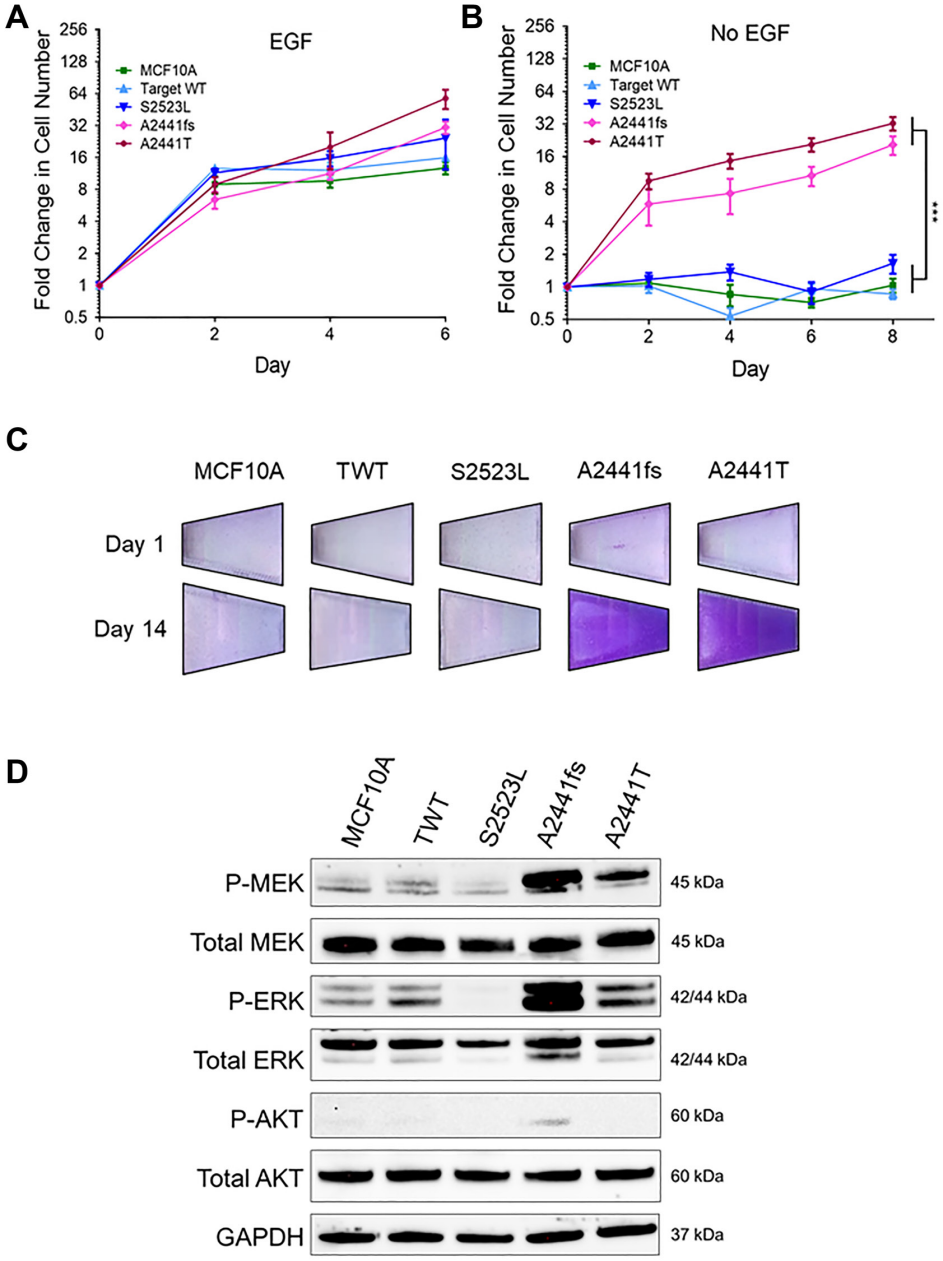
Some *NOTCH1* PEST domain variants lead to growth-factor independent proliferation. (**A**) Relative mean growth of MCF10A *NOTCH1* variant panel in the presence of physiological 0.2 ng/mL epidermal growth factor (EGF). Data are representative of the mean ± SEM (*n* ≥ 3). (**B**) Relative mean growth of MCF10A *NOTCH1* variant panel in the absence of EGF. Data are representative of the mean ± SEM (*n* ≥ 3, ^***^ *P* ≤ 0.001, 2-way ANOVA followed by Bonferroni multiple comparison test). (**C**) Representative images of EGF independent growth in the MCF10A *NOTCH1* panel stained with crystal violet on day 1 and day 14. (**D**) Immunoblot analysis of the MCF10A panel in the absence of EGF.

Increased proliferative signaling is a traditional hallmark of cancer and arguably the most fundamental trait of cancer cells [31]. Increased proliferation can be the result of a variety of processes including, but not limited to, increased signaling, loss of ligand dependence, production of growth ligands, or decreased thresholds for response [31]. To determine if *NOTCH1* variants caused increased proliferation rates in non-tumorigenic cell lines, the MCF10A and hTERT-IMEC cell line panels were grown in standard growth factor supplemented media. There was no significant difference between the *NOTCH1* variants and their controls for either the MCF10A or hTERT-IMEC panels (Figure 2A, Supplementary Figure 1A). MCF10A and hTERT-IMEC cells require epidermal growth factor (EGF) supplementation for normal proliferation. EGF is the activating ligand of EGFR, a receptor tyrosine kinase, that is often dysregulated in many malignancies. Removal of EGF from non-tumorigenic cells results in G1 arrest. Ligand-independent activation of the EGFR pathway has been associated with increased malignant potential [32]. To determine if *NOTCH1* variants impart a ligand-independent proliferative advantage, growth assays were carried out in the absence of EGF. Our results demonstrate within both *NOTCH1* panels, clones with variants at the A2441 site (A2441fs, A2441T) exhibited EGF-independent growth but the S2523L variant did not (Figure 2B, Supplementary Figure 1B). Clonogenic growth assays in the absence of EGF were carried out over 14 days and confirmed EGF-independent growth for both *NOTCH1* A2441 variants (Figure 2C, Supplementary Figure 1C).

Constitutive activation of EGFR is often the result of somatic variants, gene amplification, and/or signaling in oncogenes and leads to upregulation of the MAPK and PI3K signaling pathways [33]. To determine if either of these pathways were upregulated in the *NOTCH1* cell line panels, immunoblot analyses were carried out in the absence of EGF. For both variants at the A2441 site but not the S2523L variant, there was a significant increase in phospho-MEK and phospho-ERK, two downstream effectors of the MAPK pathway (Figure 2D, Supplementary Figure 1D). Interestingly, the A2441 variants did not have elevated phospho-AKT compared with controls, suggesting the *NOTCH1* A2441 variants activate the MAPK pathway but not the PI3K pathway.

### EGF-independent *Notch1* variants confer invasive growth phenotype and dysregulated 3D morphology

**Figure 3:**
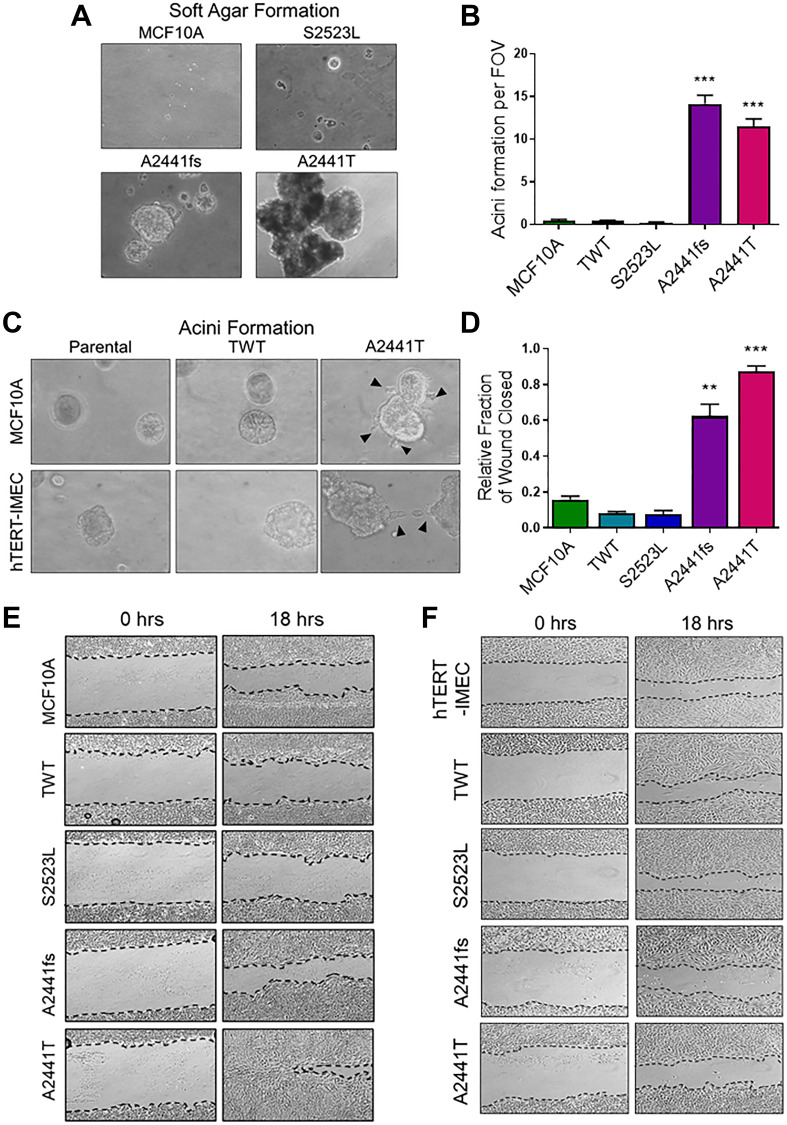
EGF-independent *NOTCH1* variants demonstrate increased transformative phenotypes. (**A**) Representative colony formation in semisolid medium cultured for 3 weeks. MCF10A cells were seeded in 0.8% soft agar plate at low density in physiologic doses of EGF (0.2 ng/mL). Magnification = 100×. (**B**) Matrigel acinar formation assay. Quantification of acini per field of view (FOV). (*n* ≥ 3, ^***^ *P* ≤ 0.001 2-way ANOVA followed by Bonferroni multiple comparison test). (**C**) Representative images of irregular morphology in the A2441T variant panel when cultured in Matrigel in the physiologic doses of EGF. Magnification = 400×. (**D**) Quantification of wound closure assay. Percentage of wound closure was measured at time of scratch (time 0) and after 18 hours. Data are representative of the mean ± SEM (*n* ≥ 3, ^**^ *P* ≤ 0.01, ^***^ *P* ≤ 0.001 2-way ANOVA followed by Bonferroni multiple comparison test). Representative images of wound closure assay of (**E**). MCF10A (**F**). hTERT-IMEC *NOTCH1* variant panel.

Anchorage independent growth in soft agar is a characteristic of cancer-associated variants and best correlates with *in vivo* tumorigenicity [34]. MCF10A cells cannot form colonies in semi-solid media or tumors in mice, however previous studies have demonstrated that aggressive genetic variants can lead to invasive colony formation in soft agar and *in vivo* tumor formation in athymic nude mice [35, 36]. In physiological doses of EGF the *NOTCH1* A2441 variants formed large, proliferative colonies, while the S2523L variants appeared to quiesce (Figure 3A). Interestingly, the A2441 variants were also capable of forming colonies in the absence of EGF (Supplementary Figure 2A and 2B). To determine if these variants affected the morphology of three-dimensional growth, acini formation assays were carried out in Matrigel. Normal MCF10A cells form uniform, hollow, acinar structures that retain important characteristic of glandular epithelium such as low proliferation and stable uniform structure [37]. In the absence of EGF, both parental cell lines and the S2523L variant were unable to form acini. However, both *NOTCH1* A2441 variants formed a significant number of colonies (Figure 3B, Supplementary Figure 2C–2E). Interestingly, in the presence of physiological doses of EGF, the A2441T variant demonstrated morphological changes in both the MCF10A and hTERT-IMEC cell lines, including protrusions and bridging suggesting loss of structural integrity and an increased transformative phenotype (Figure 3C). The ability to bridge between structures in semisolid media also suggests increased invasive and migratory potential.

### EGF-independent *Notch1* variant cells have increased migratory potential *in vitro*

Migratory capacity is associated with anchorage independent growth and increased transformative phenotypes in transformed cells. Previous studies have demonstrated Notch1 overexpression leads to increased migratory potential *in vitro* and may indicate increased metastatic potential [38, 39]. To determine if the *NOTCH1* A2441 variants demonstrated increased migratory capacity, a scratch wound assay under physiological EGF conditions was carried out in the MCF10A and hTERT-IMEC cell line panels. In the MCF10A panel, both A2441 variants demonstrated significant wound closure when compared to the WT controls and the non-phenotypic S2523L variant, (Figure 3D and 3E). Interestingly, there was no significant difference between variants and controls in the hTERT-IMEC cell line panel (Figure 3F, Supplementary Figure 3). These results suggest that variants in the A2441 variants can confer an increase in migratory potential, but that cell line context also plays a role in mediating this phenotype. Taken together with the ability to grow in semi solid media, these data suggest variants at the *NOTCH1* A2441 site may confer increased metastatic potential.

### The *NOTCH1* A2441T variant alters gene expression in cancer pathway genes

**Figure 4:**
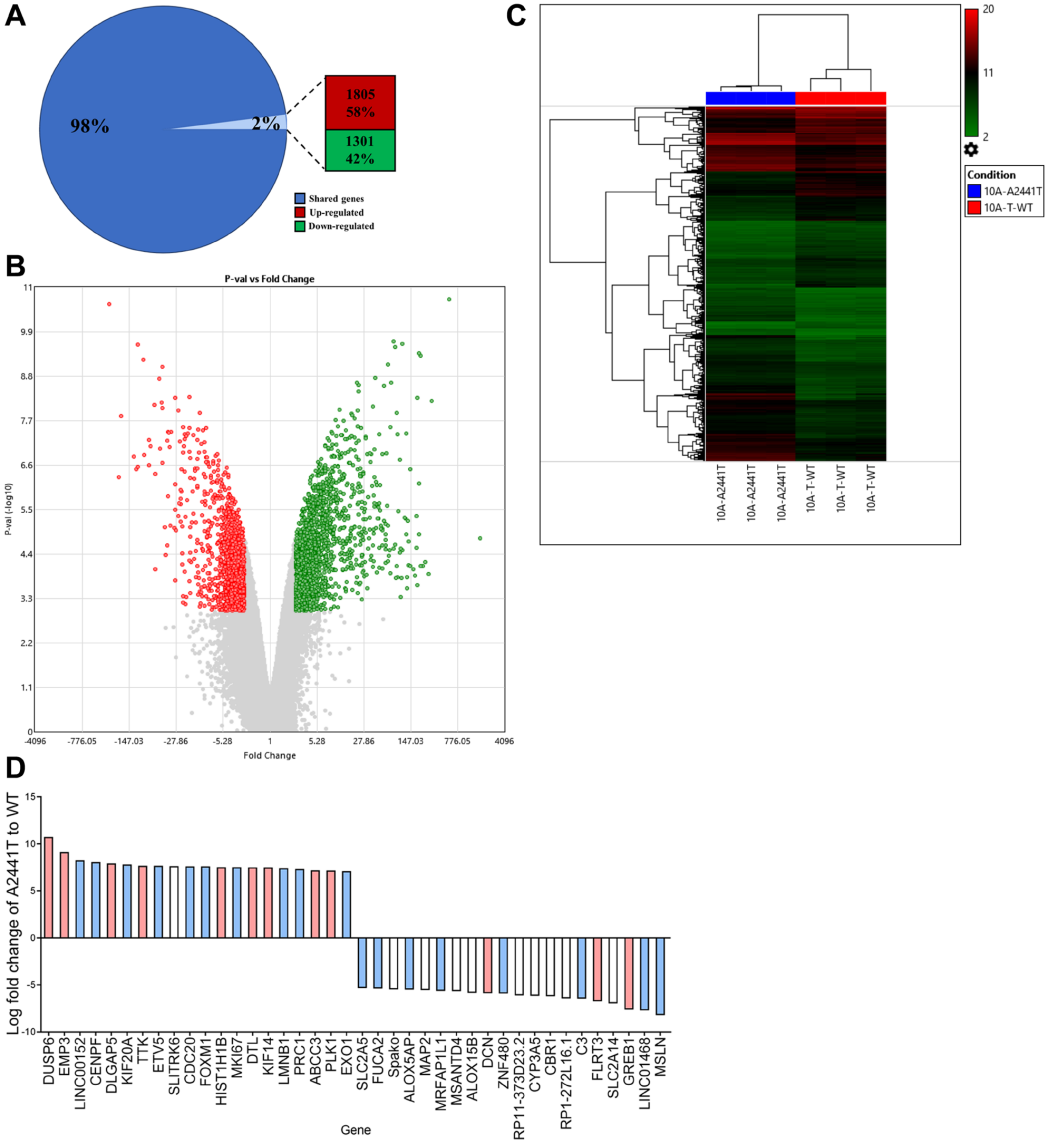
*NOTCH1* PEST variants confer gene expression changes in nontumorigenic cell lines. MCF10A A2441T variant and TWT cells were subjected to a microarray analysis with 500,000 transcripts including coding, noncoding, and splice variants. (**A**) Percentage of differentially expressed transcripts. (**B**) Volcano plot of 3106 differentially expressed transcripts (red, upregulated; green, downregulated) between A2441T and TWT. (**C**) Raw expression values (log2) for triplicate runs in each cell lines were grouped using a hierarchical clustering algorithm and are presented as a heat map (red, upregulated; green, downregulated). (**D**) The 20 genes with the highest, and 20 genes with the lowest expression in A2441T cells based on fold change. Based on a literature review, genes associated with cancer shown in blue and genes associated with breast cancer shown in red.

Notch signaling plays a fundamental role in cell differentiation and proliferation and activation of Notch signaling interacts with numerous oncogenic pathways [14]. To determine if variants at the A2441 site result in differences in gene expression, a microarray targeting over 500,000 transcripts including coding, non-coding genes, as well as exons, and splice variants (Clariom™ D human assay) was used to compare the A2441T variant to the MCF10A TWT. The A2441T site was selected due to the increased transformative properties in 3D medium. It is important to note the microarray was carried out in the presence of EGF to allow for active proliferation of both the variant and the TWT control. The microarray analysis identified 3106 differentially expressed transcripts in the A2441T cells. Out of the total number of dysregulated genes, 1805 genes were up-regulated and 1301 were down-regulated (Figure 4A and 4B). Raw expression values (log2) from three independent runs for each cell line were grouped using a hierarchical clustering algorithm and presented as a heat map (Figure 4C). The clustering confirmed the A2441T mutant cell lines demonstrated a distinct gene expression profile when compared to the TWT control. Among the 3106 differentially expressed genes, the most abundantly over and under expressed genes in A2441T were analyzed. Among the 20 genes with the highest and lowest expression, 75% of genes have been implicated in carcinogenesis (blue and pink bars, Figure 4D) and 30% of genes have been linked to breast cancer (pink bars, Figure 4D) [40]. Furthermore, we queried for genes commonly altered in TNBC [41] and found several were dysregulated in the A2441T variant (Supplementary Figure 4). There were no substantive differences in terms of MAPK signaling between A2441T knock in cells and wild type cells when grown in the presence of physiological doses of EGF. Taken together, these data suggest *NOTCH1* A2441 variants can significantly alter gene expression in non-tumorigenic breast epithelial cells.

### *NOTCH1* A2441 variants do not confer differential responses to therapies

**Figure 5:**
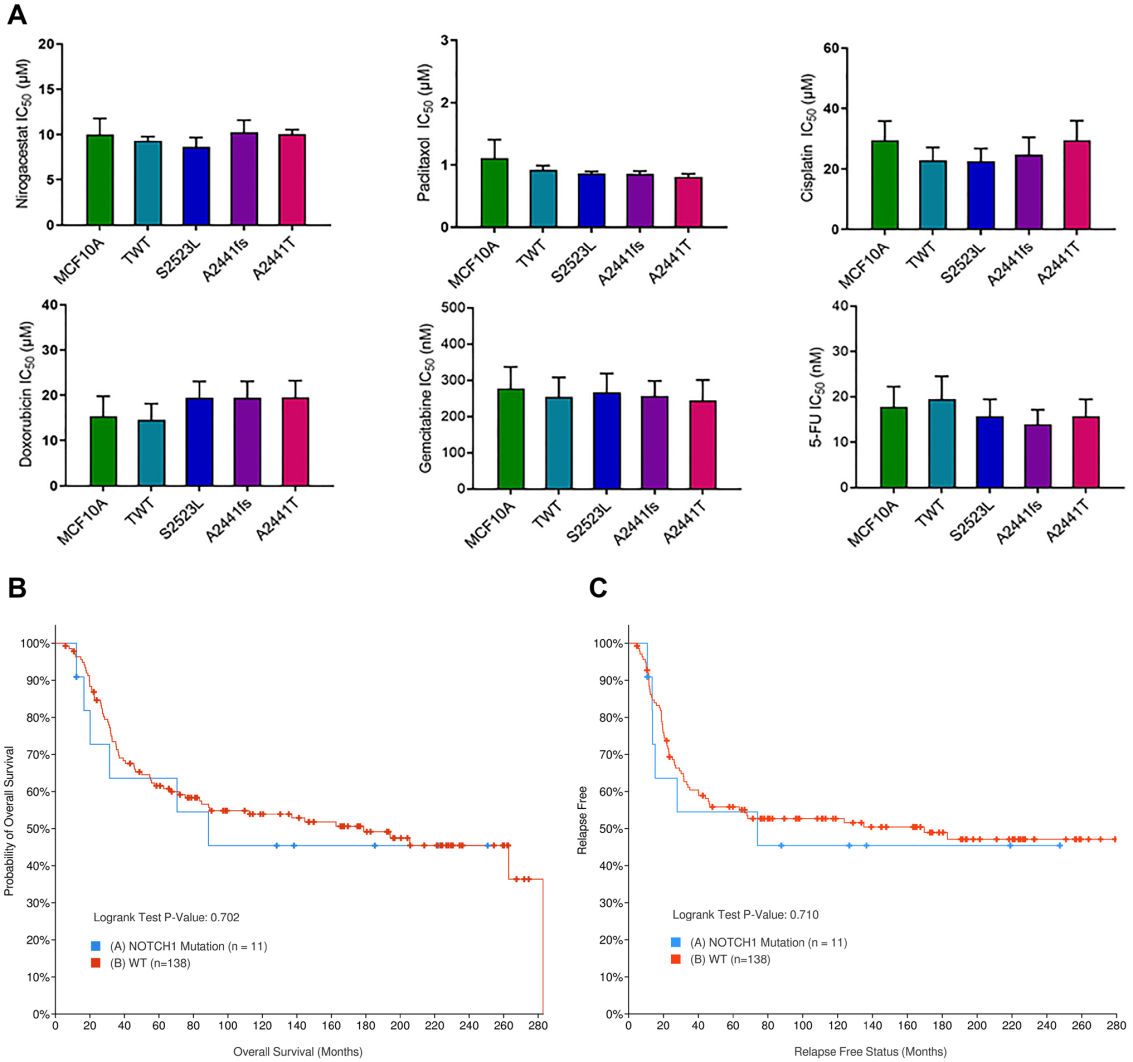
*NOTCH1* PEST variants do not demonstrate differential response to TNBC standard of care therapies. (**A**) Cell counts of the *NOTCH1* variant panel were used to determine the IC50s in 6 different standard of care therapies for TNBC (Nirogacestat, Paclitaxel, Cisplatin, Doxorubicin, Gemcitabine, and 5-FU). Data are representative of the mean ± SEM (*n* ≥ 3, ns). (**B**) Overall survival and (**C**). progression free survival in TNBC patients treated with chemotherapy with (blue) and without (red) *NOTCH1* variants. METABRIC cohort data set was analyzed via cBioPortal.

Sequencing efforts to identify potential therapeutic targets or biomarkers for TNBC has led to the distinct association of *NOTCH1* variants with TNBC. Recently, *in vitro* studies have shown GSIs preferentially target *NOTCH1* alterations and may offer a new therapeutic option for patients with TNBC [18, 42, 43]. Furthermore, in 2017 a phase I clinical trial determined the GSI nirogacestat in combination with chemotherapy was well tolerated in patients with metastatic TNBC [44]. To determine if variants at the A2441 site were specifically susceptible to GSIs or resistant to standard of care chemotherapies, IC50s for each cell line were determined for nirogacestat as well as 5 common chemotherapies. In both the MCF10A and hTERT-IMEC panels there was no observable difference in IC50s for the *NOTCH1* variants when compared to controls (Figure 5A, Supplementary Figures 5 and 6). For nirogacestat, IC50s for MCF10As and modified variants ranged between 8.6 and 10.2 μM. These ranges are considered to be on the high end of concentrations based on previous publications [15]. This may, in part, be due to the independent establishment of parental MCF10As without aberrant pathway activation. Furthermore, previous studies have demonstrated IC50s for breast cancer cell line models are significantly higher than *in vitro* concentrations for other cancer cell types [45]. Therefore, to confirm these findings in a clinical setting, we utilized the publicly available METABRIC data set via cBioPortal to determine if *NOTCH1* variants conferred a differential response to chemotherapies. Selection criteria for the comparison included patients with TNBC who received at least one chemotherapy (*n* = 149). When comparing patients with *NOTCH1* variants (*n* = 11) to patients without *NOTCH1* variants (*n* = 138), there was no significant difference in overall survival (Figure 5B) or progression free survival (Figure 5C). Although these sample sizes are small, they confirm our *in vitro* observations and suggest that despite the phenotypic changes due to the *NOTCH1* A2441 variants, they do not provide predictive value for response to chemotherapies or nirogacestat.

## DISCUSSION

This study investigated a novel *NOTCH1* PEST domain frameshift variant as well as two previously reported variants to determine cancerous phenotypes and whether these variants could serve as predictive markers for therapeutic response in TNBC. TNBC is a particularly recalcitrant disease with limited treatment options. In recent years, there has been an increasing interest in identifying molecular targets that can be utilized as potential therapeutic targets or as prognostic biomarkers [41]. These efforts have led to the identification of Notch1 as a key player in breast carcinogenesis. However, clinical studies examining the relationship between Notch1 expression and clinical outcome are inconsistent and the prognostic value of Notch1 expression remains unclear [46]. Analysis of The Cancer Genome Atlas (TCGA) database found that activated Notch1 is reportedly mutated in approximately 13% of TNBCs, is strongly enriched in the basal subtype, and is positively correlated with breast cancer progression [18]. Furthermore, studies found that activating variants in the *NOTCH1* PEST domain receptor are targetable oncogenic drivers in TNBC xenografts and are selectively responsive to GSIs [18]. However, due to the number and complexity of *NOTCH1* variants across breast cancer compared to traditional hotspot variants, classification of *NOTCH1* variants remains understudied.

Identification of transformative phenotypes within our *NOTCH1* panel revealed that both variants at the A2441 site exhibited increased MAPK pathway activation, growth factor and anchorage independent growth, and increased migratory potential. Previous studies have demonstrated that frameshift variants in the PEST domain result in increased NICD and consequently, more aggressive phenotypes. However, the A2441T variant demonstrated increased transformation in semisolid medium (Figure 3C) when compared to a frameshift at the same site. Additionally, despite also being a PEST domain variant, the S2523L variant did not exhibit any transformative properties. Taken together, these data suggest that the oncogenic potential of *NOTCH1* PEST domain variants depends on both variant type and amino acid location. Furthermore, previous studies have demonstrated the oncogenic potential of *NOTCH1* variants is heavily dependent on the type of tumor, with some cancers suggesting *NOTCH1* is an oncogene and others more consistent with a tumor suppressor gene. Our own study also shows that some phenotypes are cell type specific (increase migratory potential). The scratch wound assay showed increased closure with A2441T variants only in the MCF10A cell line but not in hTERT-IMECs. These results suggest other factors must be present or absent in hTERT-IMECs beyond the A2441T mutation and that the mutation is necessary but not sufficient to impart this phenotype. These two cell lines were derived from distinct persons, and established in different ways, with MCF10A being spontaneously immortalized while hTERT-IMECs were immortalized with hTERT overexpression. Therefore, it is not surprising that some phenotypes would be unique to one cell line. Indeed, we and others have shown in past studies differences in phenotypes and gene expression between these two cell lines [35, 47, 48]. Variability between *NOTCH1* variants as well as genomic background indicates a need for expansive classification before they can be used as reliable prognostic and/or predictive markers.

Clinical evidence has suggested *NOTCH1* variants are linked to chemotherapy resistance and emerging functional studies have suggested that Notch inhibitors, including GSIs, can effectively target *NOTCH1* variants [18, 49]. However, within our cell line panels, regardless of oncogenic phenotypes, there was no significant difference in response to GSIs or chemotherapies when compared to controls. This suggests that despite Notch pathway activation, *NOTCH1* variants are not useful predictive biomarker for treatment response and should currently not be considered when determining individual treatment courses. Despite evidence suggesting treatment resistant breast cancers can be re-sensitized by Notch inhibitors, our data provides strong rationale that further investigation is required [50]. The high variability among *NOTCH1* variants and the expansive number across the gene make it difficult to attribute therapeutic sensitivity to the presence of any single *NOTCH1* variant. This is emphasized by the lack of differential response across our panels to GSI and chemotherapies, despite demonstrating distinct phenotypes. The isogenic, non-tumorigenic background of our panels allows us to accurately assess the response to therapies for a specifically incorporated variant. Furthermore, the variants are expressed under the endogenous promoter to provide a model representative of how the variant behaves in a patient’s tumor. Future studies involving meticulous characterization of an expansive panel of *NOTCH1* variants in a similar model may provide mechanistic insight and predictive and/or prognostic value that is both variant type and site dependent.

## MATERIALS AND METHODS

### Cell culture

MCF10A cell lines were maintained in DMEM:F12 (Invitrogen) supplemented with 5% horse serum (HS, Life Technologies), 20 ng/mL epidermal growth factor (EGF, Sigma), 0.5 μg/mL hydrocortisone (Sigma), 10 μg/mL insulin (Life Technologies), 1% penicillin-streptomycin (Life Technologies), and 0.1 μg/mL cholera toxin (Sigma). hTERT-IMEC cell lines were maintained in DMEM:F12 supplemented media supplemented with 1% charcoal dextran stripped FBS (CD, Life Technologies) in place of horse serum. Parental cell lines were authenticated via short tandem repeat profiling analysis at the Johns Hopkins Genetic Resources Core Facility.

### Gene targeting and generation of *NOTCH1* variant cell lines

Gene targeting of Notch1 in MCF-10A and hTERT-IMEC cells was carried out using recombinant AAV vectors as previously described [51, 52]. Briefly, targeting vectors were designed by site-directed mutagenesis through overlap extension PCR onto a parental AAV plasmid backbone. Viral vectors were packaged using HEK-293T cells and resulting virus was transduced into targeted cell lines. Neomycin selection and PCR screening were used to select cells for homologous integration of targeting vectors via our previously described PCR-based screening method [53]. Identified colonies were single-cell diluted and selection cassettes were removed using Cre recombinase. Primer sequences for gene targeting and screening can be found in Supplementary Table 1.

### Cell proliferation assay

Cells were seeded in triplicate at 3 × 10^4^ per well and serum-starved for 24 hours before the assay media was added. Assay media consists of DMEM:F12 media containing 1% CD, hydrocortisone, cholera toxin, insulin and varying levels of EGF as indicated (no EGF or 0.2 ng/mL EGF). Medium was changed every three days and cells were counted using a Beckman Coulter counter. For crystal violet stains, cells were plated at the same density in a T25 flask, media was changed every three days, and cells were fixed and stained with 3.7% formaldehyde containing 0.2% crystal violet (Sigma).

### Colony formation assay in semisolid medium

Cells were plated at 3 × 10^3^ cells onto a 0.6% agarose layer with assay medium in a 6-well plate as previously described [54]. 0.4% agarose layer with assay medium was placed on top and changed weekly. Photographs were taken with a Nikon SMZ 1500 stereoscopic zoom microscope.

### Acinar morphogenesis assay

Morphogenesis assays were conducted in the growth factor reduced Matrigel (BD Biosciences) as previously described [54]. Cells were seeded in 8-well chamber slides containing a solid base layer of growth factor reduced Matrigel (BD Biosciences). Photographs were taken with a Nikon SMZ 1500 stereoscopic zoom microscope.

### Scratch wound healing assay

Cells were plated in six-well plates and grown under physiological growth conditions (0.2 ng/mL EGF) to near confluent monolayers. Scratch wounds were introduced in a cross pattern with a 200-μL pipette tip. Phase images and cell areas at several time points after scratch were calculated using MiToBo software as previously described [51].

### Immunoblotting

Immunoblot analysis was carried out using the NuPAGE manufacture’s protocol. The primary antibodies used in this study include: Notch1 (3608; Cell Signaling), cleaved Notch1 (4147; Cell Signaling) anti-phospho-p44/p42 MAP kinase (4370; Cell Signaling), anti-p44/p42 MAP kinase (9102; Cell Signaling), anti-phospho AKT (9271; Cell Signaling), anti-AKT (9272; Cell Signaling), anti-MEK1/2 (8727; Cell Signaling), and anti-phospho-MEK1/2 (9154; Cell Signaling). Membranes were washed and incubated with horseradish peroxidase-conjugated secondary antibodies.

### IC 50 assays

3 × 10^3^ cells were seeded in triplicate on day 0 and exposed to serial dilutions of indicated drug in media containing 0.2 ng/mL of EGF on day 1. AlamarBlue (Life Technologies) was used to determine cell proliferation according to the manufacturer’s protocol. On day 6 of assay, cells were counted using a Beckman Coulter counter and percent viability was calculated using the average cell count for each variant normalized to the appropriate DMSO control. IC50 values were calculated using the log(inhibitor) vs. response – variable slope (four parameters) nonlinear regression function in Graphpad Prism 5. All cell lines were counted in triplicate. IC50s for each variant were graphed as a function of indicated drug concentration.

### Microarray

MCF10A A2441T and TWT were propagated under normal growth conditions before RNA isolation. Gene expression profiling was performed using the Affimetrix Clariom™ D human Assay (ThermoFisher scientific), processed on the GeneChip™ 3000 by the JHMI microarray core service. Relative gene expression was determined using the log fold change (LogFC) of mutants compared to parental controls. Analyses with corresponding figures were generated using the Transcriptome Analysis Console (TAC) software (ThermoFisher scientific) specific for Clariom D assay. Classification of cancer-associated genes were confirmed by identification in previous literature [40].

### METABRIC data analysis

Analysis of the METABRIC dataset was used to compare overall survival (OS) and progression free survival (PFS) of patients with and without NOTCH1 variants when treated with chemotherapy. Within the METABRIC dataset (*n* = 2509), patients with ER-/HER- (*n* = 309) were selected from the 3-gene classifier and those who received chemotherapy (*n* = 149) were analyzed. Among these patients, 11 patients had at least 1 *NOTCH1* variant and 138 patients were classified as WT. Statistical analysis and Kaplan-Meier curves were generated by the cBioPortal algorithm. To obtain the NOTCH1 dataset, select Breast > Breast Cancer (METABRIC, Nature 2012 & Nat Commun 2016) (*n* = 2509) >3 Gene Classifier Subtypes: ER-/HER- (*n* = 309) > Chemotherapy: Yes (*n* = 149) > Mutated Genes: NOTCH1 (*n* = 11). To obtain the WT dataset utilize the custom selection to remove the NOTCH1 variants and sort for ER-/HER2- and Chemotherapy status.

### Statistics

All statistical analyses were performed using GraphPad Prism software. 2-way ANOVA tests were used to compare the experimental groups to the corresponding controls. Significance levels are indicated by one or more asterisk: ^*^ *p* ≤ 0.05, ^**^ *p* ≤ 0.01 and ^***^ *p* ≤ 0.001. Error bars represent ± SEM.

## SUPPLEMENTARY MATERIALS


