# Supplementary material for: NOTCH1 PEST domain variants are responsive to standard of care treatments despite distinct transformative properties in a breast cancer model
Source: Oncotarget. 2022 Feb 16;13:373–86. doi: 10.18632/oncotarget.28200 (PMC8849273; doi:10.18632/oncotarget.28200)
Supplement: Supplementary file 1 [file oncotarget-13-28200-s001.pdf]

NOTCH1 PEST domain variants are responsive to standard of care treatments despite distinct transformative properties in a breast cancer model

SUPPLEMENTARY MATERIALS

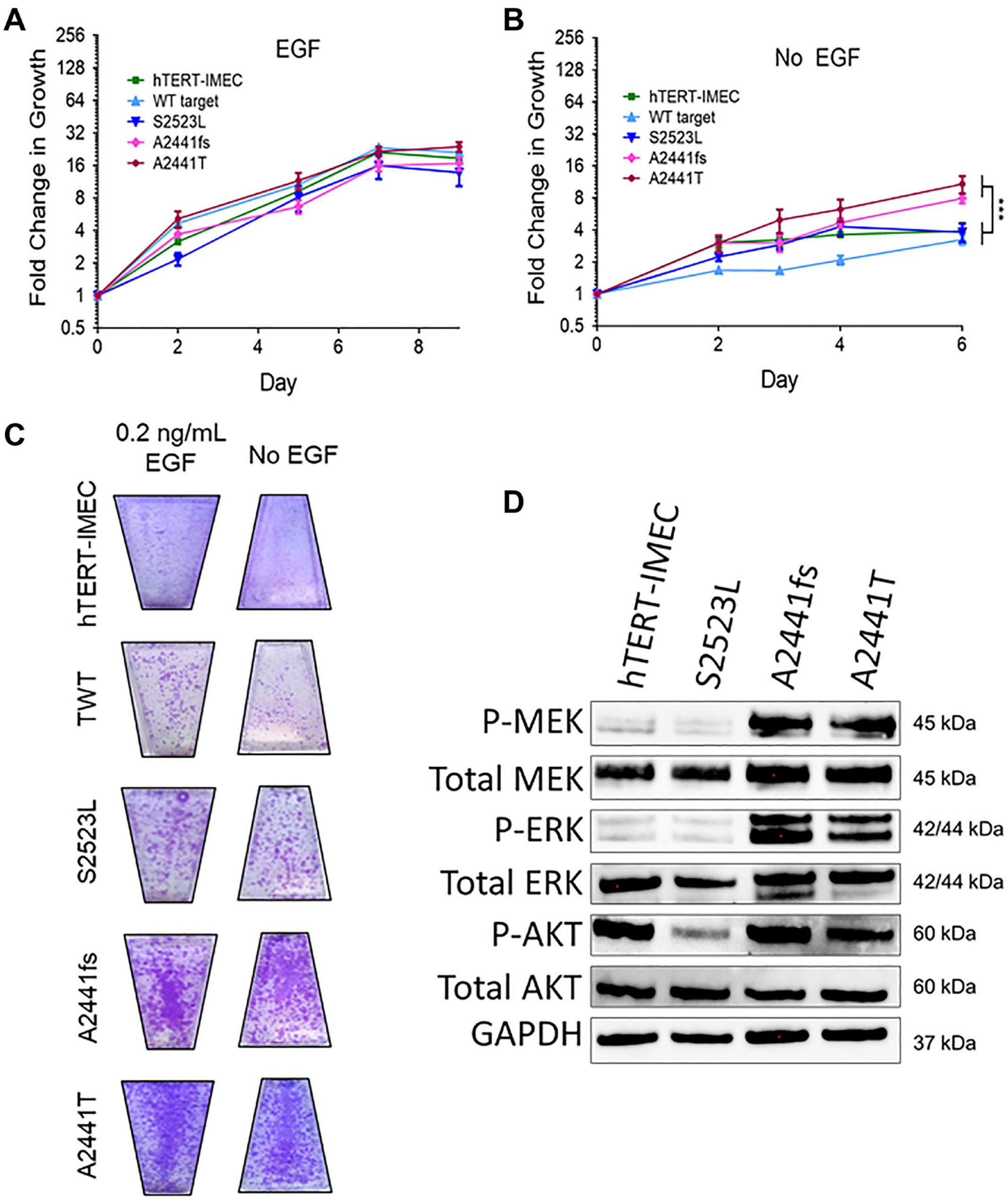

Supplementary Figure 1: A2441 *NOTCH1* variants lead to growth-factor independent proliferation in hTERT-IMECs. (**A**) Relative mean growth of hTERT-IMEC *NOTCH1* variant panel in the presence of physiological (0.2 ng/mL) epidermal growth factor (EGF). Data are representative of the mean ± SEM ($n \geq 3$). (**B**) Relative mean growth of hTERT-IMEC *NOTCH1* variant panel in the absence of EGF. Data are representative of the mean ± SEM ($n \geq 3$, $^{***}P \leq 0.001$, 2-way ANOVA followed by Bonferroni multiple comparison test). (**C**) Representative images of EGF independent growth of the hTERT-IMEC *NOTCH1* variant in physiological doses of EGF and EGF free media. Plates were stained with crystal violet. (**D**) Immunoblot analysis of the hTERT-IMEC panel in the absence of EGF.

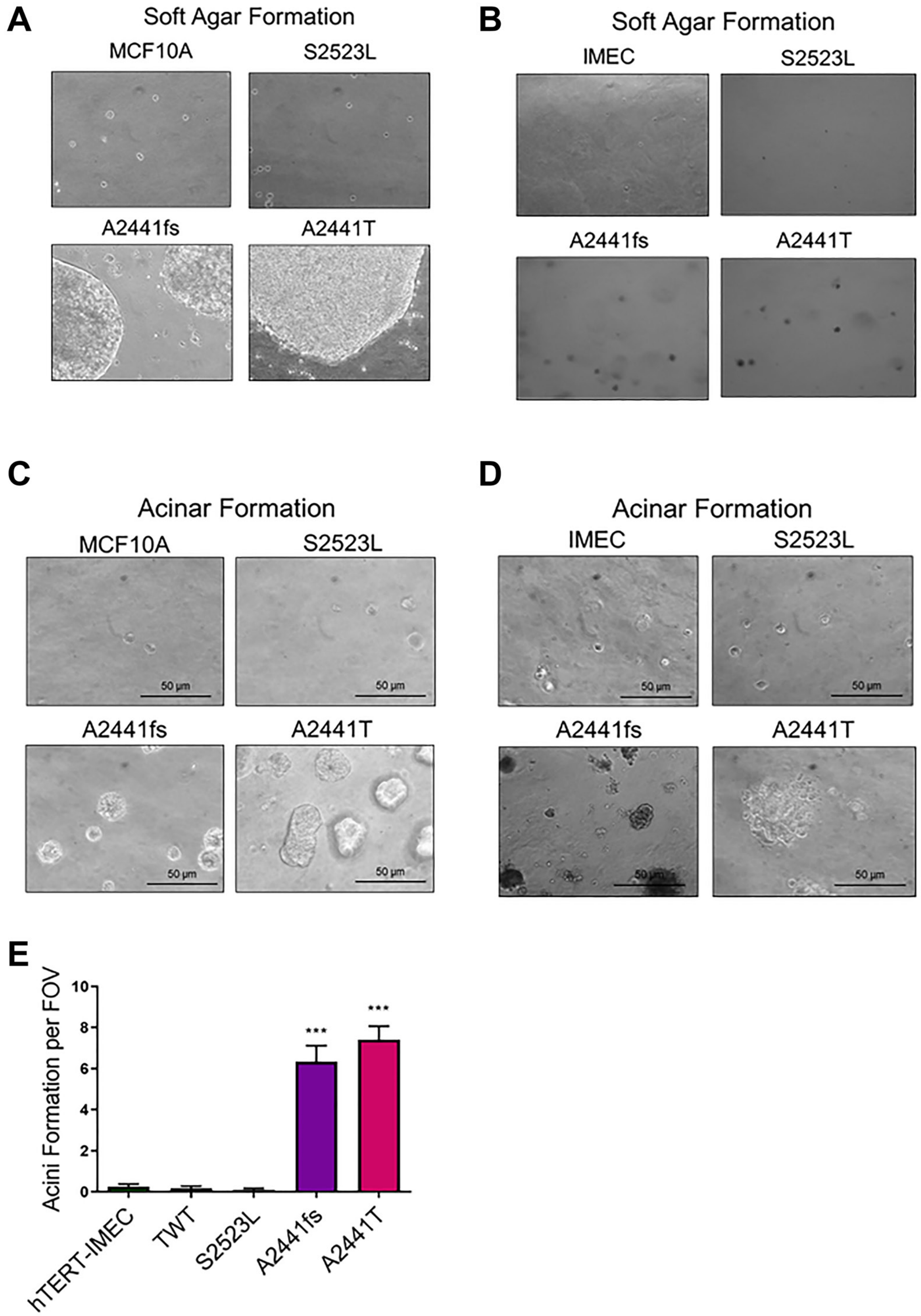

Supplementary Figure 2: A2441 *NOTCH1* variants grow in semi-solid media. (**A**) Representative colony formation in semisolid medium cultured for 3 weeks. MCF10A cells were seeded in 0.8% soft agar plate at low density in the absence of EGF. Magnification = 200×. (**B**) Representative colony formation in semisolid medium cultured for 3 weeks. hTERT-IMEC cells were seeded in 0.8% soft agar plate at low density in the absence of EGF. Magnification = 100×. Representative images of matrigel acinar formation assay for (**C**) MCF10A cells and (**D**) hTERT-IMEC cells. Bar is 50 µm. (**E**) Quantification of acini per field of view (FOV) of hTERT-IMEC assay. ($n \geq 3$, $^{***}P \leq 0.001$ 2-way ANOVA followed by Bonferroni multiple comparison test).

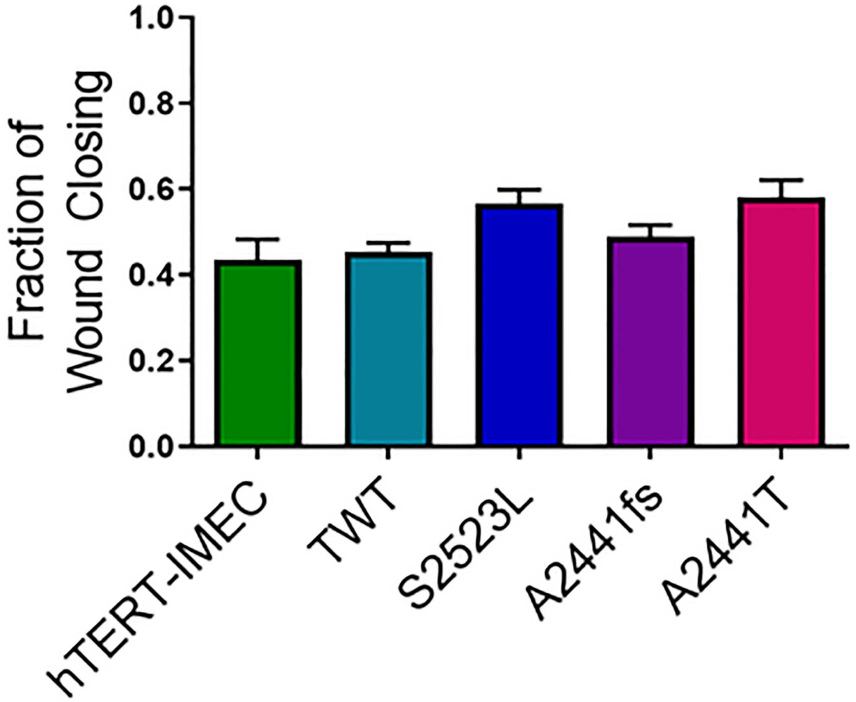

Supplementary Figure 3: hTERT-IMEC *NOTCH1* variants showed no significant difference in migration potential. Quantification of wound closure assay in hTERT-IMEC *NOTCH1* variant panel in physiologic doses of EGF. Percentage of wound closure was measured at time of scratch (time 0) and after 18 hours. Data are representative of the mean ± SEM ($n \geq 3$, ns).

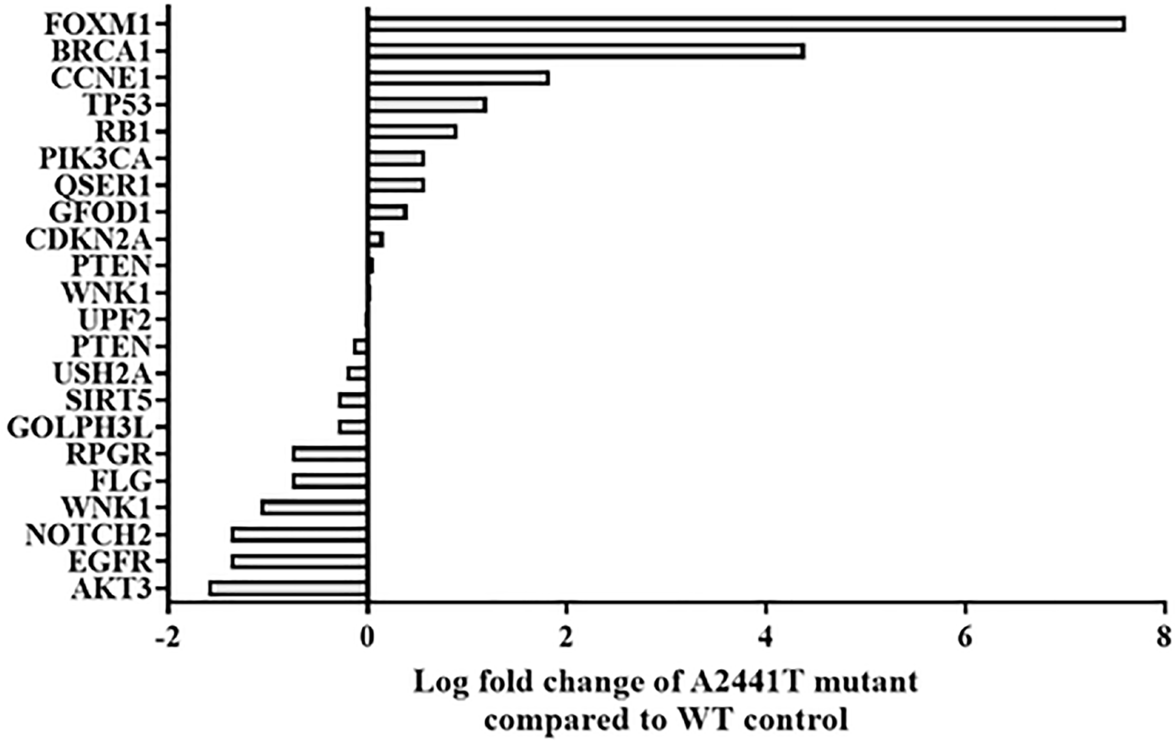

Supplementary Figure 4: The A2441T *NOTCH1* variant demonstrated dysregulation of genes associated with TNBC. The MCF10A A2441T variant and TWT cells were subjected to a microarray analysis with 500,000 transcripts including coding, noncoding, and splice variants. TNBC associate genes were identified using a previously published study [41] and graphed as a log fold change compared to TWT.

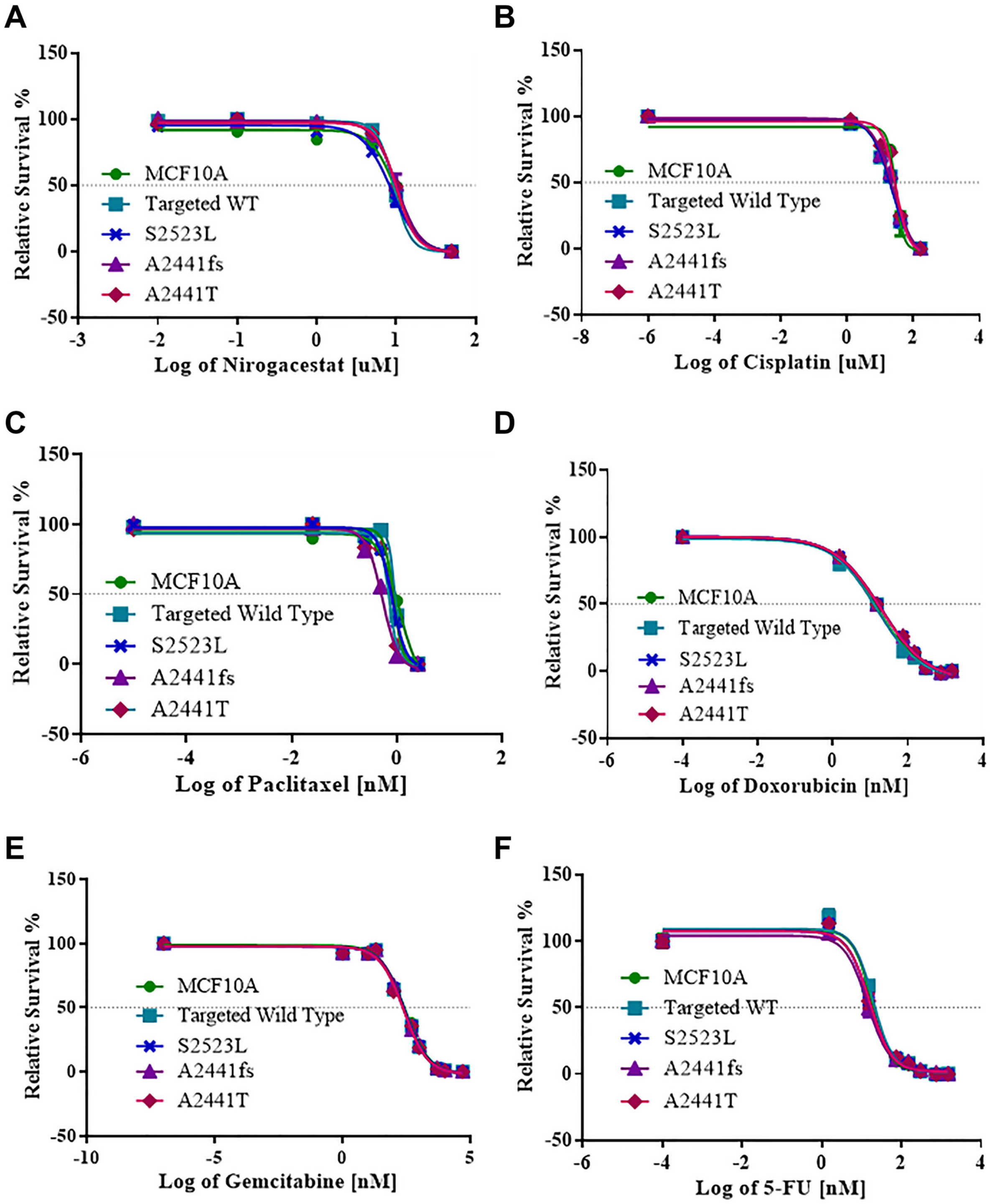

Supplementary Figure 5: IC50 curves to determine sensitivity of *NOTCH1* variants in MCF10As to TNBC standard of care therapies. Cell counts of the *NOTCH1* variant panel when exposed to varying doses of indicated therapies were used to generate IC50s in 6 different standard of care therapies for TNBC (Nirogacestat (**A**), Paclitaxel (**B**), Cisplatin (**C**), Doxorubicin (**D**), Gemcitabine (**E**), and 5-FU (**F**)). Data are representative of the mean ± SEM ($n \geq 3$, ns). IC50s were compared in Figure 5A.

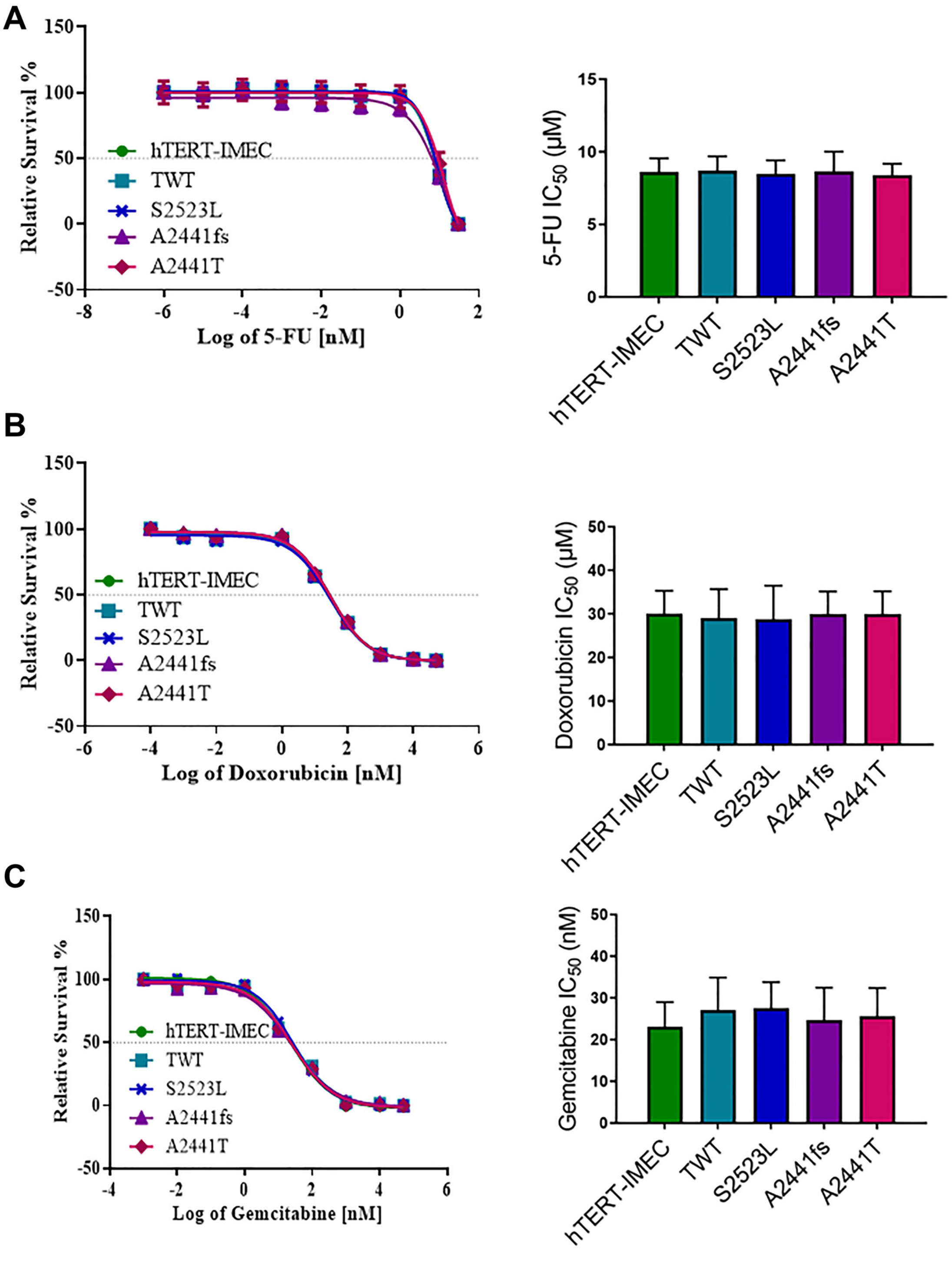

Supplementary Figure 6: *NOTCH1* variants in hTERT-IMECs do not demonstrate differential response to TNBC standard of care therapies. Cell counts of the *NOTCH1* variant panel were used to generate IC50 curves (left) and compare IC50s (right) for 3 different standard of care therapies in the hTERT-IMEC *NOTCH1* variant panel. (**A**) 5-FU, (**B**) Doxorubicin, and (**C**) Gemcitabine. Data are representative of the mean ± SEM ($n \geq 3$, ns).

Supplementary Table 1: Primers used in this study

**Homology Arm Cloning Primers**		
**Targeting Vector**	**Homology Arm**	**Forward/Reverse Primer**
NOTCH1 exon 34	5′	GTGCAGCCACAAAACTTACAGAT
		TCCGAGAACACATTTTCACAAGC
	3′	GTGTGTATGCCAAGAGTGCAC
		AAATCAACATCTTGGGACGCATC
**Pre-Cre Screening Primers**		
**Targeting Vector**	**Homology Arm**	**Forward/Reverse Primer**
NOTCH1 exon 34	5′	GGAGGGGCCCTGAATTTCAC
		GCACAGTGGTACCTTAAAATTTG
	3′	CGGGATGAGTTGGGAATAAC
		CTTGGGACGCATCTGGTCAT
**Post Cre Screening Primers**		
**Targeting Vector**	**Forward Primer**	**Reverse Primer**
NOTCH1 exon 34	AAGGCACGGAGGAAGAAGTC	CATATGCACAGTGGTACCTTAA
**Mutagenesis Primers**		
**Mutation**	**Forward Primer**	**Reverse Primer**
NOTCH1 exon34 A2441T	CTGCACGTCTGTCTGGCTCG	CGAGCCAGACAGACGTGCAG
NOTCH1 exon34 S2523L	ATGCGGGGACAAGCTGGACC	GGTCCAGCTTGTCCCCGCAT
NOTCH1 exon34 A2441fs	GAGCCAGGGAGCCAGGCAGACGTGCAGCCACTG	CCTGGCTCCCTGGCTCGGCTCTCCACTCAGG
**cDNA Sequencing Primers**		
**Targeting Vector**	**Forward Primer**	**Reverse Primer**
NOTCH1 exon34 cDNA	GCCGACCAGAGGAGCCTTTTT	ACTGGTCAGGGGACTCAGG
